# Validity of Bronchoscopist Assessment of Patient Discomfort During Flexible Bronchoscopy: An Observational Study

**DOI:** 10.7759/cureus.83331

**Published:** 2025-05-02

**Authors:** Yusuke Usui, Shion Miyoshi, Yasuhiko Nakamura, Hiroshige Shimizu, Muneyuki Sekiya, Naohisa Urabe, Takuma Isshiki, Susumu Sakamoto, Sakae Homma, Kazuma Kishi

**Affiliations:** 1 Department of Respiratory Medicine, Toho University Omori Medical Center, Tokyo, JPN

**Keywords:** benzodiazepine, bronchoscopy, discomfort, opioid, sedation

## Abstract

Background: The distress experienced by patients during bronchoscopy is often more severe than bronchoscopists anticipate. However, the use of sedatives during bronchoscopy varies depending on the facility, which may be insufficient. We examined the accuracy of bronchoscopist evaluation of predicted patient discomfort during bronchoscopy under intravenous sedation.

Methods: This study retrospectively reviewed 186 consecutive patients who underwent bronchoscopy between December 2018 and April 2019. Ultimately, 113 patients who underwent bronchoscopy with pethidine (meperidine) and 64 patients who underwent bronchoscopy with pethidine and midazolam were assigned to the pethidine group and the pethidine with midazolam combination group, respectively. We compared the patient self-assessed discomfort scores (obtained after recovery from sedation) with the discomfort scores predicted by bronchoscopists, using a five-point rating scale in each sedation group. Any discrepancy was then evaluated.

Results: The pethidine group had significantly higher self-assessed patient discomfort scores than the predictive assessment by bronchoscopists (mean score 3.4±1.3 vs. 2.9±1.2; P< 0.001). Conversely, the pethidine with midazolam group had a significantly lower self-assessed patient discomfort score than the bronchoscopists’ predictive assessment (mean score 2.4±1.5 vs. 3.3±1.4; P* <* 0.001). Multivariate analysis identified the use of midazolam as a significant factor affecting patient discomfort (odds ratio: 0.176, P < 0.001) in addition to hypoxia during bronchoscopy (odds ratio: 3.331, P = 0.008).

Conclusions: Bronchoscopists may underestimate patient discomfort during bronchoscopy under intravenous sedation. The use of midazolam was an important factor affecting the patient's discomfort during bronchoscopy.

## Introduction

Flexible bronchoscopy is a useful procedure required for diagnosis and follow-up in patients with respiratory diseases such as lung cancer, diffuse lung disease, and infection. Several guidelines have suggested the use of sedative and analgesic drugs such as benzodiazepines and opioids during bronchoscopy to relieve patient discomfort and improve tolerance [1,2]. Opioids have significant antitussive effects, and benzodiazepines have favorable sedative effects [3,4]. The British Thoracic Society (BTS) guidelines endorse a combination of opioids and midazolam with a Grade B recommendation, while short-acting agents such as fentanyl or alfentanil are suggested with a Grade D recommendation for post-procedural sedation [1]. The Japanese nationwide survey of bronchoscopy reported that 49% of facilities use intravenous sedatives routinely, while the remaining 51% do not use intravenous sedatives routinely [5]. The utilization rate of sedatives may be insufficient in Japan. Midazolam is the preferred sedative, accounting for 76.9% of all facilities, while pethidine, fentanyl, and propofol are among the other sedative and analgesic drugs utilized.

Bronchoscopists take into consideration the appropriate use of sedative drugs for each patient with regard to their general condition, age, and underlying disease. A single agent may thus be considered if there are concerns about over-sedation. Moderate sedation is considered the optimal depth of sedation and is adjusted under bronchoscopist supervision [2]. However, few reports have validated the supervision of sedation by bronchoscopists to date. The choice and dosage of sedative drugs often depend on the subjective decision of the bronchoscopist. If the bronchoscopist estimates the patient’s discomfort as mild, no additional sedative drug would be administered. In clinical practice, however, patient discomfort is sometimes more severe than the bronchoscopist anticipates. Even if the patient seems comfortable, they are often actually in considerable distress because they choose to endure the discomfort of bronchoscopy. Thus, we examined whether bronchoscopists were able to accurately estimate patient discomfort and appropriately use sedative drugs.

This study aimed to ascertain the ability of bronchoscopists to accurately assess the degree of patient distress during bronchoscopy. We investigated the discrepancy between patient discomfort during bronchoscopy as evaluated by the patients themselves subjectively after recovery from sedation and that predictively evaluated by bronchoscopists in each sedative situation. Furthermore, we investigated the effect of sedative drugs and sought to identify factors that affect patient discomfort.

This manuscript was previously posted to the Research Square preprint server on September 13, 2022.

## Materials and methods

Patients

This single-center retrospective study was conducted to evaluate the discrepancy between patient discomfort during bronchoscopy assessed by the patients themselves and by bronchoscopists and to identify factors that affect patient discomfort. We performed a retrospective analysis of patients’ discomfort data collected in clinical practice. The Ethics Committee of Toho University Omori Medical Center gave prior approval for conducting this study (approval number: M20109) according to regulations. The requirement for informed consent was waived due to the retrospective nature of the study, which was approved by the Ethics Committee of Toho University Omori Medical Center. Details about the study were disclosed on our institutional website, and the potential participants were given the opportunity to decline to be further enrolled in the study (opt-out). This study was conducted in accordance with the amended Declaration of Helsinki.

**Figure 1:**
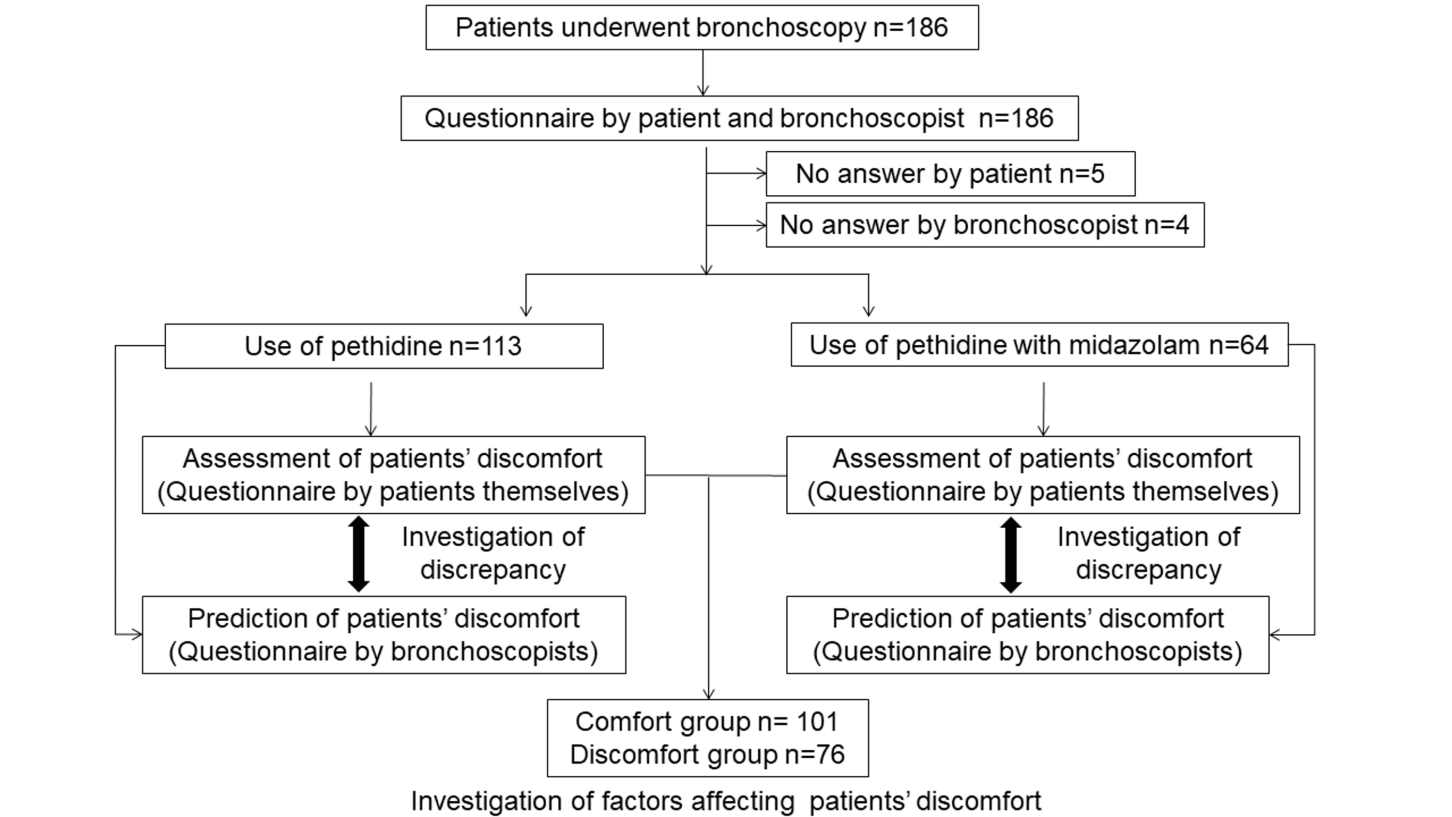
Flow diagram of patient disposition To identify factors that affect patient distress, patients were divided into 2 groups based on the rating scale for patient discomfort (question 1). Patients with 1, 2, or 3 points on the rating scale were categorized as the Comfort group, and those with 4 or 5 points were categorized as the Discomfort group.

In total, 186 consecutive patients who underwent bronchoscopy at the Toho University Omori Medical Center, Tokyo, Japan, between December 2018 and April 2019 were enrolled in this study. Nine patients were excluded because of incomplete questionnaire responses given by patients or bronchoscopists. Finally, 113 patients who underwent bronchoscopy with pethidine and 64 patients who underwent bronchoscopy with pethidine and midazolam were assigned to the pethidine and pethidine with midazolam groups, respectively (Figure 1). The choice of sedation method (either pethidine alone or pethidine combined with midazolam) was determined at the attending bronchoscopist's discretion.

Bronchoscopic procedure

In this study, we used a standard flexible bronchoscope (BF-1T260 or UC-260FW; Olympus, Tokyo, Japan) in all patients. Percutaneous oxygen saturation (SpO_2_), heart rate, and blood pressure (BP) were recorded during the examination every five minutes using electrocardiography, pulse oximetry, and BP monitoring. At the initiation of bronchoscopy, 2 L/min of oxygen was administered and subsequently titrated to maintain an oxygen saturation of at least 90%.

We performed pharyngolaryngeal anesthesia with 1% lidocaine with a Jackson-type spray prior to bronchoscopy, followed by intravenous sedation with 17.5-35 mg of pethidine. Furthermore, 2-3 mg of midazolam was combined per the decision of the bronchoscopists. Additional doses of midazolam were administered during the procedure to maintain moderate sedation based on the patient’s demeanor. The bronchoscope was introduced through the mouth. Topical anesthesia was administered by splash block with 1% lidocaine. The airway was examined, and then bronchoalveolar lavage (BAL), bronchial brushing, transbronchial biopsy (TBB), fluoroscopy-guided transbronchial lung biopsy (TBLB), and endobronchial ultrasonography-guided transbronchial needle aspiration (EBUS-TBNA) of the mediastinal lymph nodes were performed depending on the patient’s condition. After the bronchoscopic procedure, naloxone (0.2 mg) and flumazenil (0.5 mg) were administered as antagonists of pethidine and midazolam.

Complications during bronchoscopy were designated as follows: hypertension defined as an increase in systolic BP to 160 mmHg or more; hypotension defined as a decrease in systolic BP to 80 mmHg or less; and hypoxia defined as the lowest SpO_2_ of 89% or less.

Questionnaire

We developed a five-point rating visual analog scale questionnaire for the discomfort assessment (Appendix A), modified from the questionnaire developed by the Japan Society for Respiratory Endoscopy [6]. After recovery from sedation for bronchoscopy, patients underwent medical examination using a questionnaire about discomfort, memory, and pain during bronchoscopy; feelings after bronchoscopy; examination duration; and consent to re-examination. The questions were specifically (1) “Did you have any discomfort from bronchoscopy?”; (2) “What can you remember about the bronchoscopy?”; (3) “Did you feel pain during bronchoscopy?”; (4) “How did you feel after bronchoscopy?”; (5) “How did you feel about the examination duration?”; and (6) “If needed, would you consent to bronchoscopic re-examination?”. The rating scale consisted of no discomfort (1 point), almost no discomfort (2 points), normal (3 points), discomfort (4 points), and severe discomfort (5 points). Similarly, after bronchoscopy, bronchoscopists predicted patient discomfort from bronchoscopy and estimated subsequent cough and ease of bronchoscopy by using a five-point rating scale questionnaire as follows (Appendix B). Specifically (1) “How well did you anticipate the patient’s discomfort during bronchoscopy? Please give an exact estimate of the patient’s discomfort,” (2) “How severe was the patient’s cough?,” and (3) “Was the bronchoscopic examination easy to perform?.” There were no criteria provided for how the bronchoscopist determines the level of patient discomfort. The bronchoscopist referred comprehensively to the patient's vital signs, body movements, cough, and respiratory status during the examination and assessed the patient's discomfort based on the bronchoscopist's subjective assessment. We compared patient discomfort evaluated by the patients themselves (patient self-assessment score) with that evaluated by operators (bronchoscopist assessment score) and analyzed the discrepancy between them.

To identify factors that affect patient distress, patients were divided into two groups based on the rating scale for question 1. Those patients with 1, 2, or 3 points on the rating scale were categorized as the comfort group, and those with 4 or 5 points were categorized as the discomfort group.

Statistical analysis

Data are presented as median (range) or mean (range), frequencies, and percentages. Fisher’s exact test was used to compare categorical variables. Continuous variables were compared using the Mann-Whitney U test or Wilcoxon matched-pairs signed-rank test. All p-values were two-sided. A p-value less than 0.05 was considered statistically significant. Logistic regression models were constructed to estimate factors related to patient discomfort. Statistical analyses were performed using PRISM (version 8, GraphPad Software Inc., San Diego, CA, USA) and IBM SPSS Statistics for Windows, Version 22 (Released 2013; IBM Corp., Armonk, New York, United States).

## Results

The demographics of the study population are shown in Table 1. There were no significant differences between the two groups in terms of age, sex, height, weight, body mass index, frequency of bronchial washing, BAL, bronchial brushing and TBB/TBLB, rate of bronchoscopy completion, and rate of complication other than hypoxia. Procedure time was significantly longer in the pethidine with midazolam group than in the pethidine group (median (range): 25 (9-45) vs. 20 (4-45) min; P = 0.002). The pethidine with midazolam group required a higher maximum oxygen supplementation than the pethidine group (median (range): 4 (2-10) vs. 2 (2-10) L; P = 0.023). The frequency of EBUS-TBNA was significantly higher in the pethidine with midazolam group than in the pethidine group (14 of 64 (21.8%) vs. 7 of 113 (6.2%); P = 0.003). The pethidine group required a higher dose of pethidine than the pethidine with the midazolam group (median (range): 35 (17.5-35) vs. 17.5 (8.75-35) mg; P = 0.002). The rate of hypoxia was higher in the pethidine with midazolam group than in the pethidine group (20 of 64 (31.3%) vs. 20 of 113 (17.7%); P = 0.042).

**Table 1:** Demographics of the study population BMI: body mass index; BAL: bronchoalveolar lavage; TBB: transbronchial biopsy; TBLB: transbronchial lung biopsy; EBUS-TBNA: endobronchial ultrasound-guided transbronchial needle aspiration; N/A: not available; SpO₂: percutaneous oxygen saturation; COPD: chronic obstructive pulmonary disease

	Pethidine (n = 113)	Pethidine with midazolam (n = 64)	Total (n = 177)	P-value (pethidine vs. pethidine with midazolam)
Age (years, median; range)	70 (40-90)	69.5 (19-89)	70 (19-90)	0.580
Male, n (%)	76 (67.3)	33 (51.6)	109 ((61.6)	0.053
Height (m, median; range)	1.64 (1.34-1.81)	1.59 (1.44-1.81)	1.61 (1.34-1.81)	0.143
Weight (kg, median; range)	57 (34.3-107.8)	58.2 (32.8-83)	57.7 (32.8-107.8)	0.847
BMI (kg/m^2^, median; range)	21.4 (14.4-35.2)	22.6 (15.1-31.1)	21.8 (14.4-35.2)	0.184
Procedure time (min, median; range)	20 (4-45)	25.0 (9-45)	22 (4-45)	0.002
Maximum oxygen supplementation (L, median; range)	2 (2-10)	4 (2-10)	2 (2-10)	0.023
Procedures				
Bronchial washing, n (%)	90 (79.6)	45 (70.3)	135 (76.3)	0.198
BAL, n (%)	34 (30.1)	17 (26.6)	51 (28.8)	0.730
Brushing, n (%)	60 (53.1)	31 (48.3)	91 (51.4)	0.639
TBB/TBLB, n (%)	53 (46.9)	35 (54.7)	88 (49.7)	0.350
EBUS-TBNA, n (%)	7 (6.2)	14 (21.8)	21 (11.9)	0.003
Dose of sedative given				
Pethidine (mg, median; range)	35 (17.5-35)	17.5 (8.75-35)	17.5 (8.75-35)	<0.001
Midazolam (mg, median; range)	N/A	2 (1-7)	0 (0-7)	N/A
Completion of bronchoscopy, n (%)	106 (93.8)	60 (93.8)	166 (93.8)	>0.999
Complications, n (%)				
Pneumothorax	0	1 (1.6)	1 (0.6)	0.362
Hypertension	55 (48.7)	28(43.8)	83 (46.9)	0.536
Hypotension	2 (1.8)	0	2 (1.1)	0.536
Hypoxia	20 (17.7)	20 (31.3)	40 (22.6)	0.042
Coexisting conditions, n (%)				
COPD	23 (20.3)	8 (12.5)	31 (17.5)	0.221
Interstitial pneumonia	36 (31.9)	20 (31.3)	56 (31.6)	>0.999
Indication for bronchoscopy, n (%)				
Lung tumor	48 (42.5)	33 (51.6)	81 (45.8)	0.274
Interstitial lung disease	32 (28.3)	17 (26.6)	49 (27.7)	0.862
Infectious disease	24 (21.2)	10 (15.6)	34 (19.2)	0.430
Other	9 (8.0)	4 (6.3)	13 (7.3)	0.772

Results of patient-assessed as well as bronchoscopist-assessed patient parameter scores using a visual analog scale are shown in Table 2. Regarding patient self-assessment, the pethidine with midazolam group had significantly milder discomfort than the pethidine group (mean score 2.4±1.5 vs. 3.4±1.3; P < 0.001). Memory during bronchoscopy was clearer (mean score 4.7±0.8 vs. 2.9±1.5; P < 0.001), and examination time was perceived as longer (mean score 2.7±1.3 vs. 1.8±1.0; P < 0.001) by patients in the pethidine group than in the pethidine with midazolam group. Pain during bronchoscopy was mild in both groups but slightly milder in the pethidine with midazolam group than in the pethidine group (mean score 1.2±0.7 vs. 1.6±1.2; P = 0.036). Nevertheless, there were no differences between the two groups in terms of consent to re-examination (mean score 2.8±1.5 vs. 2.9±1.5; P = 0.554) and feeling after bronchoscopy (mean score 3.1±1.2 vs. 2.8±1.0; P = 0.077). Regarding the bronchoscopists’ assessment, the predicted patient discomfort during bronchoscopy was significantly more severe in the pethidine with midazolam group than in the pethidine group (mean score 2.9±1.2 vs. 3.3±1.4; P < 0.027). There were no differences between the two groups in terms of cough (mean score 2.7±1.3 vs. 3.1±1.5; P = 0.091) and ease of bronchoscopy (mean score 2.4±1.2 vs. 2.6±1.6; P = 0.532).

**Table 2:** Self-assessed and bronchoscopist-assessed patient parameter scores using a visual analog scale Data are shown as mean±standard deviation.

Parameter	Pethidine group n = 113	Pethidine with midazolam group n = 64	p-value
Patient-assessed score			
Discomfort during bronchoscopy (score)	3.4±1.3	2.4±1.5	<0.001
Memory during bronchoscopy (score)	4.7±0.8	2.9±1.5	<0.001
Pain during bronchoscopy (score)	1.6±1.2	1.2±0.7	0.036
Feeling after bronchoscopy (score)	3.1±1.2	2.8±1.0	0.077
Duration of examination (score)	2.7±1.3	1.8±1.0	<0.001
Consent to re-examination (score)	2.8±1.5	2.9±1.5	0.554
Bronchoscopist-assessed score			
Prediction of patient discomfort during bronchoscopy (score)	2.9±1.2	3.3±1.4	0.027
Cough (score)	2.7±1.3	3.1±1.5	0.091
Ease of bronchoscopy (score)	2.4±1.2	2.6±1.6	0.532

**Figure 2:**
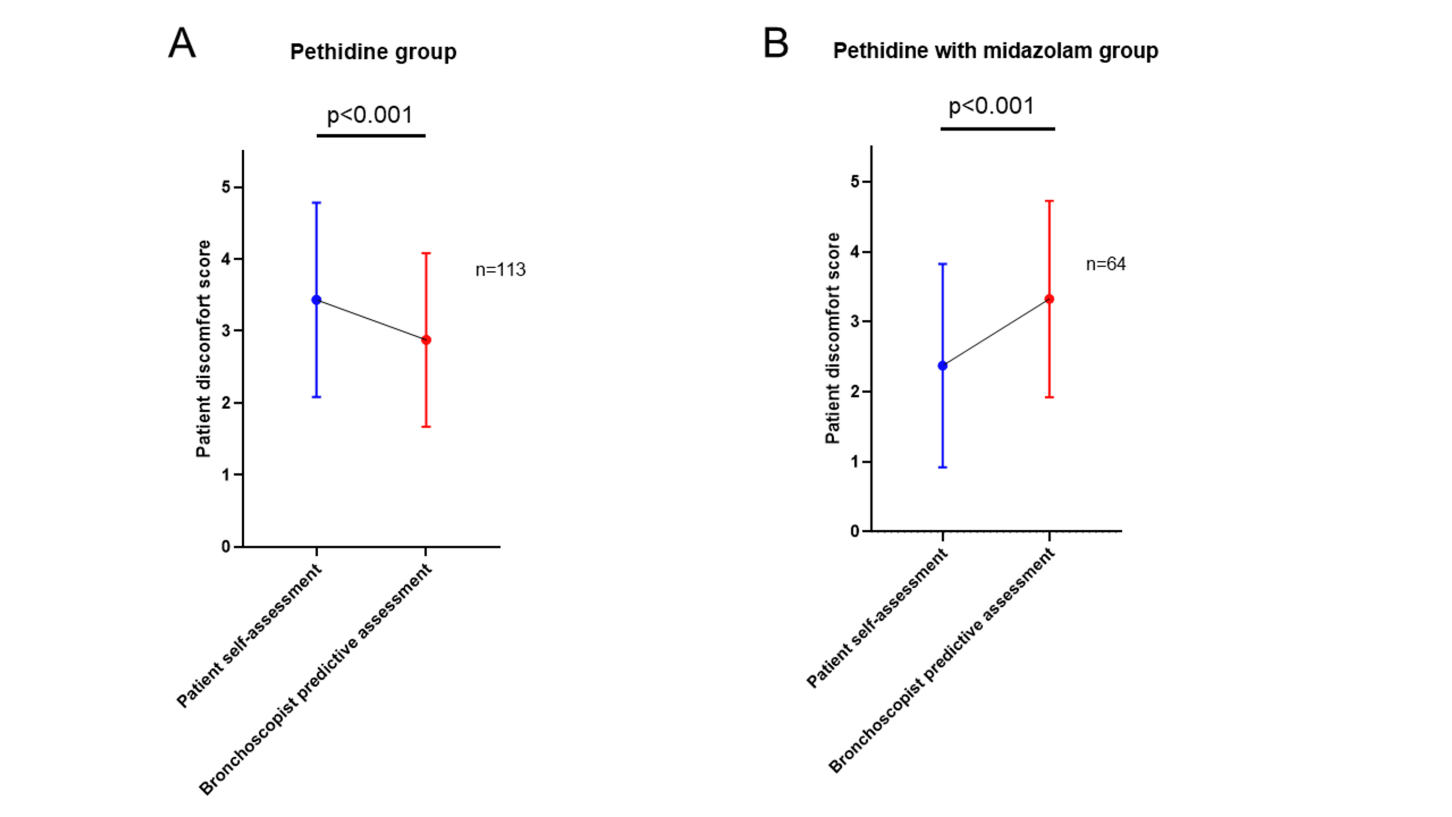
Comparison of discomfort scores in each sedation group between patient self-assessment and bronchoscopist predictive assessment (A) In the pethidine group, the discomfort score by patient self-assessment was significantly higher than by bronchoscopist predictive assessment (P < 0.001). (B) In the pethidine with midazolam group, the discomfort score by patient self-assessment was significantly lower than by the bronchoscopist predictive assessment (P < 0.001).

A comparison of patient discomfort scores in each sedation group between the patient self-assessment and the bronchoscopist predictive assessment is presented in Figure 2. The pethidine group had significantly higher patient discomfort scores by patient self-assessment than by bronchoscopist predictive assessment (mean score 3.4±1.3 vs. 2.9±1.2; P < 0.001) (Figure 2A). Conversely, in the pethidine with midazolam group, this was significantly lower than by patient self-assessment than by bronchoscopist predictive assessment (mean score 2.4±1.5 vs. 3.3±1.4; P < 0.001) (Figure 2B).

**Figure 3:**
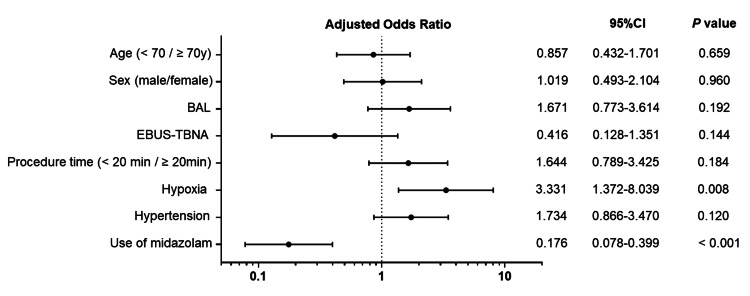
Forest plot of adjusted odds ratio in multivariable analysis of factors affecting patient discomfort score by patient self-assessment The horizontal axis displays the logarithmic scale. BAL: bronchoalveolar lavage; EBUS-TBNA: endobronchial ultrasonography-guided transbronchial needle aspiration

Next, we compared the discomfort and comfort groups to evaluate factors affecting patient discomfort (Table 3). Univariate analysis revealed a significantly higher rate of midazolam use in the comfort group than in the discomfort group (50% (50 of 101) vs. 18% (18 of 76); P < 0.001). In multivariate analysis using a logistic regression model, the use of midazolam was identified as a significant factor affecting patient discomfort (odds ratio: 0.176, P < 0.001) in addition to hypoxia (odds ratio: 3.331, P < 0.008) (Figure 3).

**Table 3:** Univariate analysis for discomfort score BMI: body mass index; BAL: bronchoalveolar lavage; TBLB: transbronchial lung biopsy; EBUS-TBNA: endobronchial ultrasonography-guided transbronchial needle aspiration; SpO₂: arterial oxygen saturation of pulse oximetry; BP: blood pressure

	Discomfort group (n = 76)	Comfort group (n = 101)	p-value
Age, < 70 years, n (%)	35 (46)	47 (47)	> 0.999
Sex, male, n (%)	47 (62)	62 (61)	> 0.999
BMI, < 22 kg/m^2^, n (%)	45 (59)	47 (47)	0.128
Procedure			
Bronchial washing, n (%)	57 (75)	78 (77)	0.726
BAL, n (%)	27 (36)	24 (23)	0.096
Brushing, n (%)	38 (50)	53 (52)	0.763
TBB/TBLB, n (%)	39 (51)	49 (49)	0.762
EBUS-TBNA, n (%)	5 (7)	16 (16)	0.065
Procedure time, ≥ 20 min, n (%)	45 (59)	60 (59)	> 0.999
Hypoxia, n (%)	23 (30)	17 (17)	0.455
Hypertension, n (%)	40 (53)	43 (43)	0.073
Use of midazolam, Yes, n (%)	14 (18)	50 (50)	<0.001

## Discussion

This study highlighted two important clinical issues. First, the discrepancy in patient discomfort was investigated using patient self-assessment and bronchoscopist predictive assessment. Second, regardless of the prediction by bronchoscopists, the use of midazolam in addition to pethidine relieved patient discomfort during bronchoscopy.

The purpose of this study was to evaluate the validity of bronchoscopist assessment of patient discomfort during bronchoscopy. Therefore, we investigated the discrepancy between patient discomfort during bronchoscopy assessed by patients themselves and that assessed by bronchoscopists. The results demonstrated significant discrepancies in both the pethidine and the pethidine with midazolam groups. The pethidine group had significantly higher patient discomfort than bronchoscopists anticipated. This implies that bronchoscopists underestimated patient discomfort during bronchoscopy in the pethidine group. However, in the pethidine with midazolam group, patient discomfort was significantly lower than bronchoscopists anticipated, implying, in this case, that they overestimated patient discomfort during bronchoscopy in the pethidine with midazolam group. Based on these findings, we conclude that it was difficult for bronchoscopists to accurately estimate patient discomfort during bronchoscopy regardless of the type of sedative drug.

In this study, therefore, the discrepancy between the discomfort score by patient self-assessment and by bronchoscopist predictive assessment differed between the two groups. In the pethidine-alone group, the dose of pethidine was higher than in the pethidine and midazolam combination group (Table 1). Thus, it follows that bronchoscopists might have underestimated patient discomfort levels because of the strong antitussive effect of pethidine, which suppressed the cough. In contrast, bronchoscopists overestimated patient discomfort levels in the pethidine and midazolam combination group. This combination group had a lower pethidine dose than the pethidine-alone group (Table 1), which may have contributed to the fact that cough was slightly less suppressed and patients seemed more distressed (Table 2). Bronchoscopists need to pay attention to such behavioral modifications in patients by the sedative drugs and should not rely solely on the prediction of patient discomfort.

A few previous studies have shown that bronchoscopists are unable to accurately assess patient satisfaction during the bronchoscopy [7,8]. There is also a report that the estimation of patient pain by endoscopists and endoscopy assistants during colonoscopy is often inaccurate [9]. It would be difficult for the endoscopist to accurately predict the level of patient discomfort during endoscopic examination. In our study, bronchoscopists were unable to accurately predict the patient's level of discomfort. Furthermore, they either overestimated or underestimated it depending on the type of sedative used. Therefore, interventions to reduce the patient's discomfort during the bronchoscopy may need to be based on decisions other than the operator's subjective evaluations.

The use of midazolam in addition to pethidine relieved patient discomfort during bronchoscopy in this study. The patient self-assessed discomfort score was significantly lower in the pethidine and midazolam combination group than in the pethidine alone group (Table 2). Furthermore, in multivariate analysis, the use of midazolam was a significant factor in improving patient self-assessed discomfort scores. It is known that patients are less distressed and more tolerant of bronchoscopy under benzodiazepine sedation than with placebo [7,10]. Benzodiazepines alone had a better sedative effect than opioids alone, while opioids alone had a better antitussive effect than benzodiazepines alone [11,12]. Therefore, the combination of these two drugs may be a reasonable modality to compensate for the shortcomings of the other. Topical anesthesia with lidocaine is also recommended to help the patient tolerate the procedure [1]. To reduce the risk of lidocaine toxicity, bronchoscopists should be cautious about using excessive lidocaine. Dexmedetomidine has the potential to be a useful drug for bronchoscopy because it causes bronchodilation and prevents tachycardia [13].

It is important to note that patient tolerance to anesthetic drugs is highly variable, requiring individualized attention during bronchoscopy. While sedation should ideally be titrated for each patient based on their specific characteristics and responses, this remains challenging in clinical practice. Currently, no optimal mode of proper dosage adjustment for sedative drugs during bronchoscopy has been established. It is difficult to adjust the dose of midazolam by subjectively inferring patient distress levels during bronchoscopy because bronchoscopists cannot accurately estimate patient discomfort for reasons discussed in the previous paragraphs. Thus, consideration may be given to dose-adjustment methods other than the bronchoscopists’ predictive assessment. Several studies have been conducted to determine the proper dosage of midazolam using bispectral index monitoring [14-16]. One of these studies reported the optimal depth of sedation during bronchoscopy as a Ramsay sedation score of 4, which is a brisk response to light, glabella tap, or loud noise [16,17]. While these reports about the depth of sedation are useful, further studies on the optimal dose are needed.

To date, few reports have examined the safety of the combination of benzodiazepine and opioid [18-21]. In our study, no serious complications occurred in either group, but the lowest SpO2 was slightly lower in the combination group (Table 2); this was managed by administering supplemental oxygen. Also, previous reports described deep sedation occurring frequently during endoscopy with combined opioid and benzodiazepine sedation [22,23]. It is possible that the combination of these two drugs causes unexpectedly deeper sedation than with single agents. However, there was ultimately no difference in the completion rate of the bronchoscopic procedure between the two groups in this study (Table 1). This suggests that there were no serious complications caused by sedative drugs. Thus, adding midazolam to pethidine seems to be safely manageable and reduces patient discomfort. In low-risk patients, it appeared that pethidine alone should be avoided, and midazolam should be used in combination with pethidine based on patient discomfort exceeding that predicted by the bronchoscopist. Multivariate analysis revealed that hypoxia worsened patient discomfort, with higher rates observed in the pethidine with midazolam combination group. Notably, despite experiencing more hypoxia events, patients in this combination group reported less discomfort overall. This finding suggests that midazolam makes a significant contribution to reducing patient discomfort during the procedure. It appears that the potent sedative effects of midazolam are strong enough to override the discomfort associated with hypoxia.

Several guidelines recommend sedation with midazolam for bronchoscopy procedures [1,2]. At our institution, the combination of midazolam is considered, taking into account patient anxiety, age, overall health status, and comorbidities. In this retrospective study, the decision to use midazolam in combination or not was based on the subjective judgment of the bronchoscopists, and the selection criteria were not clearly defined. As a result, the combination group had a higher proportion of EBUS-TBNA procedures (Table 1). American College of Chest Physicians guidelines recommend deeper sedation for EBUS-TBNA procedures, as this technique involves manipulating the respiratory tract, which can trigger coughing reflexes and patient discomfort [24]. Additionally, the number of cases in the combination group was smaller compared to the pethidine group. There is a possibility that some bronchoscopists, due to their limited familiarity with the use of midazolam, expressed concerns about respiratory depression associated with the combination regimen.

There were several limitations in our study. First, this was a retrospective study conducted in a single center, leading to possible bias in patient selection. Nevertheless, we were able to include almost all consecutive patients who underwent bronchoscopy in our hospital during the study period. In addition, we did not evaluate delirium or anterograde amnesia in the midazolam combination group, which are known complications of benzodiazepines. This oversight may have affected our assessment of discomfort scores, as amnesia could influence patients' recall of procedural discomfort. Second, the bronchoscopic procedures were heterogeneous. Different variations of the procedure may affect the level of patient discomfort differently. Third, this was a non-blinded study; hence, a possible bias in the discomfort score as assessed by the patient and bronchoscopist cannot be ruled out. Further prospective blinded randomized control trials are needed to evaluate the discomfort score without bias.

## Conclusions

There was significant discrepancy between patient self-assessment and bronchoscopist assessment of patient discomfort during bronchoscopy under sedative administration. It was difficult for bronchoscopists to accurately estimate patient discomfort regardless of the type of sedative drug; therefore, bronchoscopists should proactively consider sedation with combined benzodiazepine and opioid to reduce patient discomfort and improve tolerance during bronchoscopy.

## Figures and Tables

**Figure 4:**
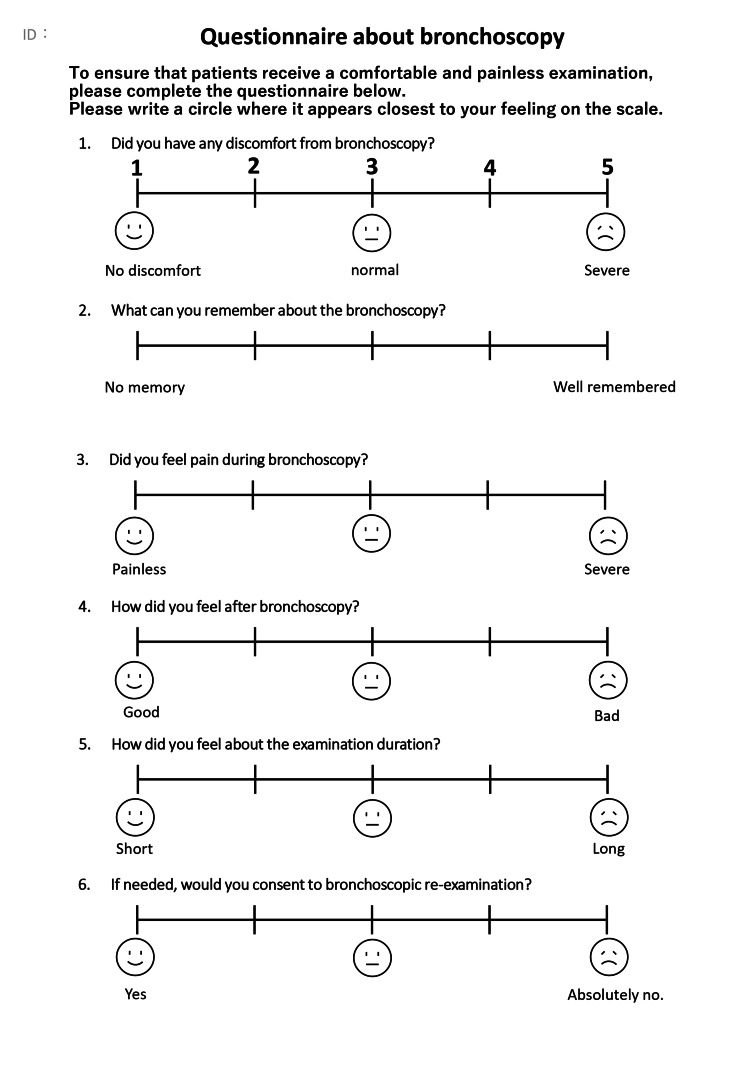
Questionnaire about bronchoscopy for patient Questionnaire for the discomfort assessment. The rating scale consisted of no discomfort (1 point), almost no discomfort (2 points), normal (3 points), discomfort (4 points), and severe discomfort (5 points).

**Figure 5:**
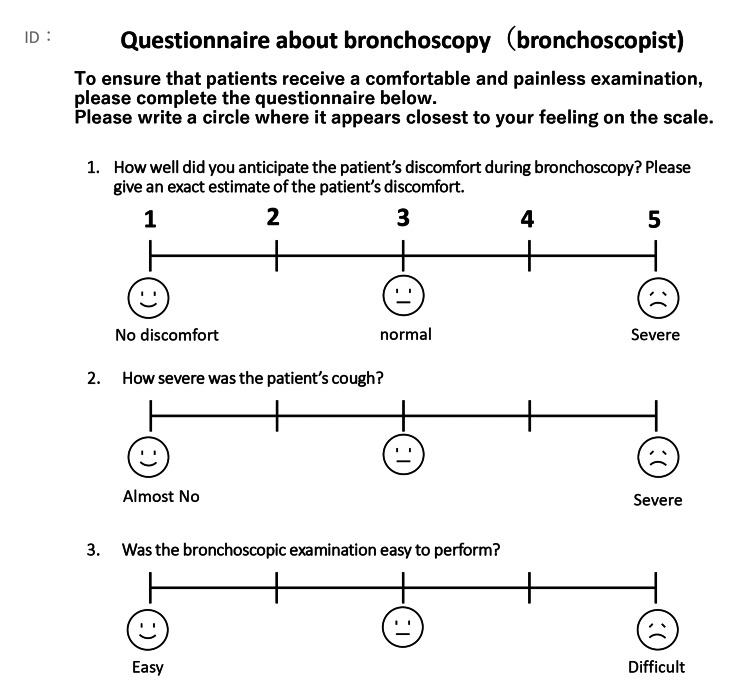
Questionnaire about bronchoscopy for bronchoscopist Questionnaire for the discomfort assessment. The rating scale consisted of no discomfort (1 point), almost no discomfort (2 points), normal (3 points), discomfort (4 points), and severe discomfort (5 points).

## Appendices

 Appendix A

 Appendix B
